# Exploring Adaptive Virtual Reality Systems Used in Interventions for Children With Autism Spectrum Disorder: Systematic Review

**DOI:** 10.2196/57093

**Published:** 2024-09-18

**Authors:** Luna Maddalon, Maria Eleonora Minissi, Thomas Parsons, Amaia Hervas, Mariano Alcaniz

**Affiliations:** 1 Laboratory of Immersive Neurotechnologies Institute Human-Tech Universitat Politècnica de València Valencia Spain; 2 Grace Center, Edson College Arizona State University Tempe, AZ United States; 3 Computational Neuropsychology and Simulation Arizona State University Tempe, AZ United States; 4 Child and Adolescent Service, University Hospital Mutua Terrassa Global Institute of Neurodevelopmental Disorders Integrated Care University of Barcelona Barcelona Spain

**Keywords:** adaptive system, virtual reality, autism spectrum disorder, intervention, training, children, machine learning, biosignal

## Abstract

**Background:**

Adaptive systems serve to personalize interventions or training based on the user’s needs and performance. The adaptation techniques rely on an underlying engine responsible for processing incoming data and generating tailored responses. Adaptive virtual reality (VR) systems have proven to be efficient in data monitoring and manipulation, as well as in their ability to transfer learning outcomes to the real world. In recent years, there has been significant interest in applying these systems to improve deficits associated with autism spectrum disorder (ASD). This is driven by the heterogeneity of symptoms among the population affected, highlighting the need for early customized interventions that target each individual’s specific symptom configuration.

**Objective:**

Recognizing these technology-driven therapeutic tools as efficient solutions, this systematic review aims to explore the application of adaptive VR systems in interventions for young individuals with ASD.

**Methods:**

An extensive search was conducted across 3 different databases—PubMed Central, Scopus, and Web of Science—to identify relevant studies from approximately the past decade. Each author independently screened the included studies to assess the risk of bias. Studies satisfying the following inclusion criteria were selected: (1) the experimental tasks were delivered via a VR system, (2) system adaptation was automated, (3) the VR system was designed for intervention or training of ASD symptoms, (4) participants’ ages ranged from 6 to 19 years, (5) the sample included at least 1 group with ASD, and (6) the adaptation strategy was thoroughly explained. Relevant information extracted from the studies included the sample size and mean age, the study’s objectives, the skill trained, the implemented device, the adaptive strategy used, the engine techniques, and the signal used to adapt the systems.

**Results:**

Overall, a total of 10 articles were included, involving 129 participants, 76% of whom had ASD. The studies included level switching (7/10, 70%), adaptive feedback strategies (9/10, 90%), and weighing the choice between a machine learning (ML) adaptive engine (3/10, 30%) and a non-ML adaptive engine (8/10, 80%). Adaptation signals ranged from explicit behavioral indicators (6/10, 60%), such as task performance, to implicit biosignals, such as motor movements, eye gaze, speech, and peripheral physiological responses (7/10, 70%).

**Conclusions:**

The findings reveal promising trends in the field, suggesting that automated VR systems leveraging real-time progression level switching and verbal feedback driven by non-ML techniques using explicit or, better yet, implicit signal processing have the potential to enhance interventions for young individuals with ASD. The limitations discussed mainly stem from the fact that no technological or automated tools were used to handle data, potentially introducing bias due to human error.

## Introduction

### Background

Recent research on assessment, training, and intervention applied to technologies has focused on creating complex adaptive systems [1-5]. According to Almirall et al [6], an adaptive intervention is characterized by a set of clinical decision rules that offer guidance on when and how to adjust the dosage and nature of the treatment, considering specific measures. In this way, adaptivity can be defined as the system’s capability to alter its actions in response to the preferences and needs of the user [4,7]. Moreover, adaptive systems have the potential to enhance the individualized training experience and prevent issues such as overtraining, undertraining, cognitive overload, frustration, and boredom [5]. This differs from the nonadaptive approach, where the same settings are used throughout training, or adjustment is based on settings that are unrelated to the participant’s performance [8].

Technologies, such as robots, mobile phones, and screen-based systems, provide controlled and engaging environments that facilitate adaptive training and support the development of multiple interaction abilities in a secure and predictable manner [9]. The conventional computer is the predominant hardware choice for adaptive and personalized systems [3,4] because adaptive systems have been built upon existing development tools and infrastructure designed for traditional computers and devices. This approach aims to streamline the development process, ultimately reducing the human effort and time required for implementation. In contrast with traditional computer-based therapies, virtual reality (VR) excels in promoting ecological validity by delivering immersive, lifelike experiences, thus creating a strong sense of presence and facilitating the transfer of learning outcomes to real-world situations [10-13]. VR is intended as a computer-generated simulation of an environment that allows users to interact with and experience an artificial world as if it were real. VR aims to create a sense of presence, where users feel as though they are physically present within the virtual environment, enabling them to explore the simulated space, manipulate objects, and engage with the surroundings in a lifelike manner [12]. Indeed, VR technology can achieve varying degrees of immersion, categorized into 3 levels: nonimmersive, semi-immersive, and fully immersive (depending on the capabilities of the device being used). VR offers various avenues for adaptation, including adjusting the complexity of content, tailoring evaluations, and modifying autonomous virtual agents [14]. Another form of adaptation could involve integrating system input, such as using voice commands and haptic feedback. Moreover, VR not only enables the recording of real-time information but also facilitates the integration of data collected from various devices, each dedicated to monitoring distinct psychophysiological activities [15]. Several studies have yielded significant training findings on implementing adaptive VR interfaces. Among them are interventions related to mental health or neuropsychiatric conditions, such as emotional and affective training [16], treatment of phobias [17], management of pathological stress [18], and therapy for posttraumatic stress disorder [19].

In recent decades, research has focused on using VR in the assessment, treatment, and training of neurodevelopmental disorders such as autism spectrum disorder (ASD) [13,20-25]. Studies increasingly focus on this disease due to the escalating worldwide incidence and the high demand for early interventions [26]. Moreover, extensive research has demonstrated that training with VR can lead to notable enhancements in various domains among the young population with ASD [14,21,23-25]. ASD is a neurodevelopmental condition characterized by impairments in social communication and the presence of restrictive and repetitive behaviors [27]. One distinguishing aspect of ASD is its spectrum nature, indicating significant variability in symptom severity among the individuals affected. This heterogeneity results in clinical phenotypes that differ substantially from one person to another while sharing common underlying features. The heterogeneity in symptom severity observed in ASD requires the implementation of personalized treatment approaches that target each individual’s specific symptom configuration. By designing early interventions tailored to each child’s characteristics, it becomes possible to provide targeted training and improvements in deficits commonly associated with ASD [14,28]. In this connection, adaptive VR technologies seem to have the potential to pave the path for a new generation of highly efficient technology-driven therapeutic tools for the young population with ASD.

Following this approach, a variety of ASD adaptive technologies have been proposed. Among them, Bian et al [29] presented a VR training system for improving driving skills that autonomously adapted its difficulty levels according to participants’ engagement and performance metrics. Another adaptive system was applied to a VR job interview training platform, which dynamically adjusted the conversation according to users’ responses and stress levels [30]. Research has demonstrated that using such a solution for rehabilitation yields favorable outcomes in enhancing certain abilities of children with ASD; specifically, the VR system was able to tailor the intervention in accordance with the user’s actions [31].

Given the evidence demonstrating the effectiveness of using adaptive VR systems to address deficits associated with ASD and the showcased advantages of early intervention, there arises a need to examine the technical aspects of system adaptation and the complexities of handling user data. To do this, it is crucial to dissect the components of an adaptive system generally used to train or treat ASD symptoms. The following taxonomy will facilitate the reading of this work.

### Adaptive Strategy: Level Switching, Feedback, and Time

A system can be adapted through different strategies, such as level-switching techniques or feedback. Level switching follows a logic of level difficulty, and the choice of switching can be based on progression or regression techniques. The progression technique can be considered the core training principle because it is necessary to increase the difficulty level to continue training certain skills, improve them, and prevent the occurrence of learning effects. Therefore, in this scenario, training is adapted through a gradual increase in difficulty level based on the individual’s abilities. Unlike progression, the regression technique allows for a finer, more flexible, and more nuanced approach, adapting the difficulty level according to the arising needs. Rooted in traditional therapy practices, this strategy allows for dynamic adjustments that increase or decrease the difficulty level based on the observed performance of the individual [8]. This would ensure that the individual is able to perform satisfactorily at an easier level before increasing the difficulty level, allowing more time to learn the content [8].

Conversely, feedback consists of a system response transmitted visually, audibly, verbally, or haptically to enhance user understanding. Both strategies can occur in 2 temporal dimensions: real time or deferred time. Real-time adaptation occurs based on real-time measures during training, while deferred adaptation happens before the training or session begins, and it depends on the trainee’s prior knowledge, level, preferences, or experience [5]. Underlying these, an engine is responsible for processing the information it receives (refer to the Signals: Explicit and Implicit subsection) to transmit an ad hoc response regarding level switching and feedback. The role of the engine can be performed either by a professional, resulting in person-automatized technologies; or directly by the system itself, resulting in system-automatized technologies.

### The Adaptive Engine: Person-Automatized Technologies and System-Automatized Technologies

Within the engine’s framework, it is defined as person automatized when a professional is in charge of analyzing the engine input signals and making decisions about the type of adaptation needed according to each case. Often, clinicians adapt the interaction by observing the user without playing any active role in the virtual environment [28]. Alternatively, users may interact with a virtual environment where the main avatars are controlled by real people who adapt the system responses according to the user [32,33]. Although this is a valid choice for adaptation, the limitation lies in the need for technical expertise to effectively use these systems [32]. In person-automatized systems, a professional makes clinical decisions by integrating behavioral and psychophysiological user data to adapt the system. However, this could result in time and bias problems. Conversely, advanced systems that automate clinical integrations through multicomputer interactions stand out as a promising choice [32].

A cost-effective and streamlined approach involves using a system-automatized adaptive engine [5]. In this configuration, the system autonomously processes input signals and directly makes decisions to tailor the intervention. Thereby, together with reducing the signal processing time and adaptation decision delays, there is a lower risk of errors or attention problems for professionals [9,34]. In this context, non–machine learning (ML) techniques can be strategically used to adapt the engine. These techniques rely upon explicitly defined rules or heuristics to guide decision-making and system adaptation. These rules are typically designed by professionals and are based on a set of predetermined conditions and actions. The process involves analyzing input signals and making adaptive decisions based on the application of rule-based methods, handcrafted algorithms, or expert knowledge [35,36]. Engines can also be designed through ML techniques to make adaptive decisions based on learned patterns from data rather than rigid explicit rules. ML models are trained on stored data to make predictions or decisions. These systems then refine their knowledge through data-driven methodologies, automated learning processes, and continuous improvement mechanisms, enabling them to learn and make strategic adaptive decisions autonomously [29,30].

In recent years, the focus has been moving increasingly toward system-automatized technologies that use real-time adaptive techniques [34,37]. Indeed, these adaptive systems are capable of responding in real time to behaviors with accuracy beyond human capabilities [34]. Real-time responses seem to confer advantages in creating effective and realistic interventions [35,38]. To enhance user experience, comfort, or task efficiency, systems may apply ML techniques to recognize patterns in the data, allowing them to make adaptive decisions in real time [29,39]. This is often achieved by using sensors and algorithms to interpret and analyze the data effectively by leveraging the input signals of the system.

### Signals: Explicit and Implicit

Concerning what has been reported about adaptive strategies and their features (level switching, feedback, time, and engine), it is now important to explain what kind of information is processed by the engine to make an adaptive decision. A signal refers to user information or data used as input to the engine that guides the adaptive decision-making. The adaptation process occurs through the recording, analyzing, and interpreting of the collected signals to make informed decisions about how the system should adjust its behavior, settings, or functionality. On the basis of the available information, the underlying goal is to improve the system’s performance, efficiency, efficacy, and user experience. These signals can be explicit or implicit in nature, and they play a crucial role in enabling the system to adapt and respond effectively to changing conditions. Only through the analysis of the user’s signals can an intervention be adapted according to the user’s needs. The signal is considered explicit when it corresponds to visible behaviors, such as verbal responses or task performance. The latter typically involves quantifiable metrics of how effectively a user is performing a specific task, with the purpose of informing the system about the quality or efficiency of the task execution [40,41]. By contrast, the implicit signal (or biosignal) refers to data or information inputs that are not explicitly provided by a user but are inferred or recorded by the system. These implicit biosignals often include nonverbal behaviors such as eye gaze, motor movements, speech patterns, and peripheral physiological responses [30,36,42,43]. Implicit biosignals are valuable because they can uncover insights not directly visible from the user’s overt actions.

Moreover, real-time processing of behavioral, biological, and psychological information is used to continuously assess the user’s state and adapt the human-machine interface. Adaptive VR systems establish robust communication with users, detecting their current state and adjusting tasks to support specific behavioral goals.

### Aim of the Study

In accordance with the aforementioned points, in recent years, the use of adaptive VR systems as a therapeutic tool for individuals with ASD has garnered increasing attention [20,23-25]. Due to the diverse nature of ASD, which manifests differently in individuals and evolves over the lifespan, intervention requirements differ significantly between pediatric and adult populations. Acknowledging the substantial evidence endorsing the advantages of early intervention [44], this systematic review concentrates specifically on the pediatric population affected by ASD. Therefore, considering the relevance of using adaptive systems in ASD to improve the customization of treatment based on young individuals’ characteristics and their progress, this systematic review primarily aims to comprehensively investigate and analyze the existing literature on the application of adaptive VR systems in the context of interventions for children with ASD. Specifically, the goal is to discuss those studies that have implemented adaptive VR systems, analyzing the study objectives, the methodologies chosen for the functioning of the adaptive engine, and the types of signals implemented. This review focuses only on system-automatized adaptive engines due to their convenience compared with the person-automatized ones [5,32]. Therefore, the methodology investigated refers to level switching, feedback, time, the autonomous adaptation of system engines, and the signals processed. In addition, this review aims to identify the current body of research on adaptive VR systems used in interventions for children with ASD, highlight methodological considerations, and propose directions for future research to guide the development and refinement of adaptive VR interventions for young individuals on the autism spectrum.

To our knowledge, there are no systematic reviews or meta-analyses on this topic. The only similar work is the study by Alcañiz et al [9], which, however, did not use a systematic approach for the comprehensive screening and review of existing literature. Furthermore, it not only considered those adaptive interventions in VR but followed a broader gaze aimed at any type of technology.

## Methods

### Approach

The literature search adhered to the PRISMA (Preferred Reporting Items for Systematic Reviews and Meta-Analyses) guidelines [45], ensuring a rigorous search process for this systematic review. The PRISMA checklist is available in Multimedia Appendix 1.

### Literature Search Strategy

A comprehensive search was conducted on March 13, 2024, to identify relevant studies in 3 different databases: PubMed Central, Scopus, and Web of Science. These databases were searched using a combination of keywords and Medical Subject Headings terms related to the research question; in addition, the literature was searched for peer-reviewed articles and conference articles in English, with full texts available, published between January 1, 2013, and March 13, 2024. Considering the recent increase of interest in the field and the continual evolution of hardware and software, a literature review that includes sources published in approximately the past decade would provide an up-to-date overview of the state of the art, ensuring the inclusion of the most advanced technologies and the latest methodologies [23,46,47]. The first author used the following Boolean string to search the databases: (“virtual” OR “VR”) AND (“adaptive” OR “personalized” OR “customized” OR “individualized” OR “tailored”) AND (“training” OR “intervention” OR “therapy” OR “clinical*”) AND (“toddler” OR “children” OR “infant” OR “teen*” OR “young adult*”) AND (“autistic spectrum disorder” OR “autism” OR “ASD”). Studies that fulfilled the following inclusion criteria were selected: (1) the experimental tasks were presented through a VR system, (2) the adaptation was system automatized, (3) the VR system was designed for intervention or training of ASD symptoms, (4) participants’ ages varied between 6 and 19 years, (5) the sample included at least 1 group with ASD, and (6) the adaptive strategy was comprehensively explicative. Accordingly, the following criteria were used to exclude studies from this systematic review: (1) studies not using a VR system for experimental tasks, (2) absence of automated adaptation mechanisms within the VR system, (3) studies whose objective was not specifically aimed at intervention or training of ASD symptoms, (4) studies involving participants outside the age range of 6 to 19 years or absence of specified age, (5) absence of at least 1 group with ASD in the study sample, and (6) studies failing to provide a sufficient or clear explanation of the adaptive strategy used. Applying these criteria aimed to ensure the selection of studies meeting stringent standards for relevance to the review objectives and rigor in methodology.

The selection of studies was divided into 6 stages, as outlined in Textbox 1.

**Study selection stages.**

**Stages involved in the selection of studies**

- Stage 1: the first author conducted the literature search of the databases and manually removed duplicates. The data were recorded in a template to ensure consistency in the data collection process and organized for subsequent analysis. Following the PRISMA (Preferred Reporting Items for Systematic Reviews and Meta-Analyses) recommendations to avoid bias and reduce errors, the 5 authors operated independently from the second stage onward.
- Stage 2: the titles and abstracts of the retrieved studies were reviewed to identify potentially eligible ones. Reasons for excluding studies were documented.
- Stage 3: a full-text assessment based on predefined inclusion and exclusion criteria was performed. Any discrepancies between the reviewers were resolved through discussion and consensus. Reasons for excluding studies were documented.
- Stage 4: the quality of the selected studies was assessed using the JBI critical appraisal tool [48] to identify and evaluate the risk of bias. Each article was evaluated according to standardized criteria, such as study design, the robustness of the findings, and potential sources of bias.
- Stage 5: relevant data from each included study were systematically collected using a predefined data extraction form. Relevant information categories extracted from the studies were chosen and defined by the 5 authors following the aim of this systematic review.
- Stage 6: the extracted data were combined and analyzed to provide an overall summary of the evidence. The data synthesis involved a narrative synthesis that was tabulated to facilitate the further interpretation of the findings.

No technological or automated tool was used to manage the collected records. Relevant information extracted from the studies included the sample size and mean age, the objectives of the study, the skill trained, the implemented device, the adaptive strategy used, the engine chosen, and the signal used to adapt the systems. The adaptive strategy findings are classified by the mode used to adapt the intervention, such as through level switching (progression or regression) and feedback, the time when adaptation occurs (real time or deferred time), and the engine executing the adaptation (ML based or non-ML based). Feedback can be verbal, auditive, visual, or haptic. The signals reported in the results refer only to those feeding the adaptive engine. Signals recorded for subsequent analyses that did not refer to the adaptive strategy were excluded.

### Ethical Considerations

This study was deemed exempt by the Polytechnic University of Valencia.

## Results

### Review Flow

**Figure 1:**
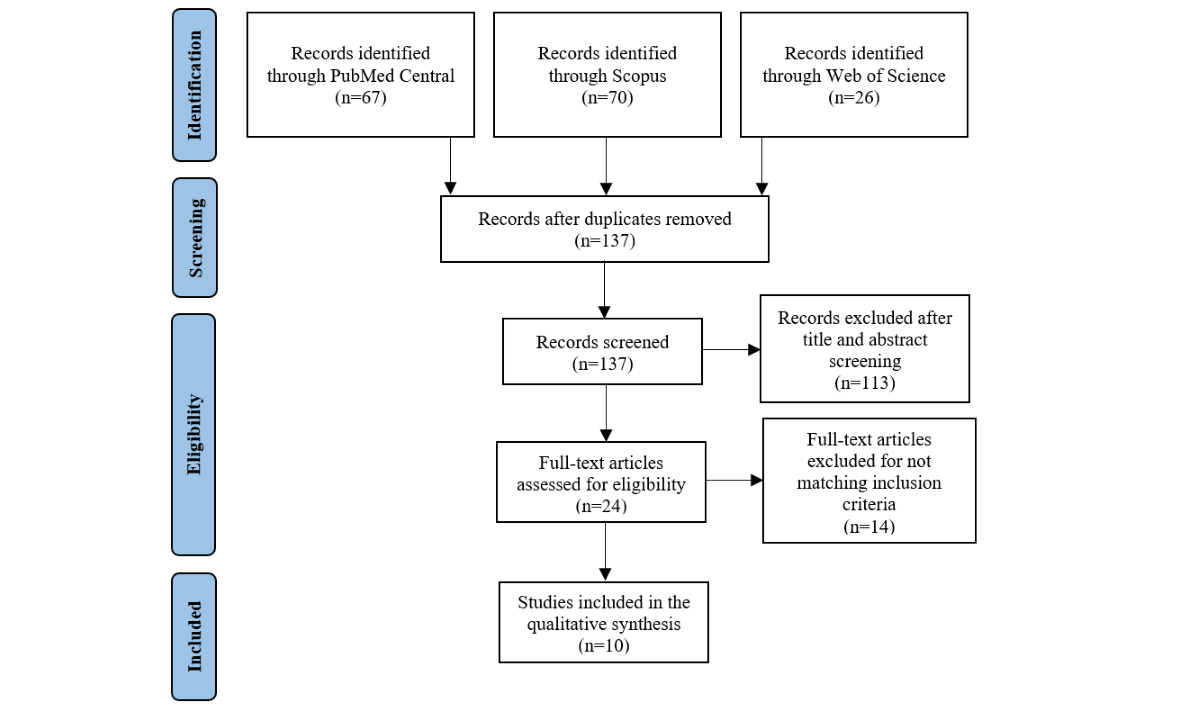
Flow diagram of the study selection process.

The study selection process was documented using a PRISMA flow diagram (Figure 1). The search strategy we used yielded 163 records (PubMed Central: n=67, 41.1%; Scopus: n=70, 42.9%; Web of Science: n=26, 16%). From the 163 articles, 26 (16%) duplicates were removed, leaving 137 (84%) unique articles. Of these 137 articles, 113 (82.5%) were excluded based on title and abstract screening. Of the remaining 24 articles, 14 (58%) were excluded after a full-text screening, which was performed to ensure that the studies met the inclusion criteria. Some of the studies (9/14, 64%) initially seemed to meet the inclusion criteria; however, subsequent analysis revealed discrepancies leading to their exclusion; for instance, some of the studies (3/9, 33% 9/14, 64%%) were excluded due to the implementation of person-automatized systems rather than system-automatized processes as required [32,33], while some of the articles (6/9, 67%) were excluded because they lacked an experimental design [49]. Finally, 10 (6.1%) of the initially identified 163 articles satisfied the inclusion criteria and were thus included in the review. The data collected were analyzed following a systematic approach used to identify and synthesize findings.

### Characteristics of the Selected Studies

The selected studies are presented in Table 1 in alphabetical order. The 10 studies involved 129 participants, of whom 95 (73.6%) were children with ASD. The mean ages of the participants ranged from 8 to 16 years (mean 12.54, SD 2.12 y). With regard to study objectives, of the 10 studies, 6 (60%) were usability studies [36,38,40,42,50,51], 3 (30%) had a further objective aimed at treatment [35,37,41], and 1 (10%) aimed merely at treatment. All included studies designed training through serious games, thus having the purpose of teaching or educating through a game. Most of the studies (7/10, 70%) focused on training social skills [35,36,40,42,43,50,51], while 20% (2/10) concentrated on motor skills [37,38], and 10% (1/10) targeted executive functions [41]. The devices used were desktop computers (8/10, 80%) [35,36,38,40-43,50], a head-mounted display (1/10, 10%) [37], and a tablet computer (1/10, 10%) [51].

Regarding the adaptive strategy, level switching was based either on a progressive strategy (4/10, 40%) [37,41,50,51] or a regressive strategy (3/10, 30%) [36,40,42]. Moreover, the systems provided adaptive feedback through the use of verbal (8/10, 80%) [36,38,40-43,50,51], audio (4/10, 40%) [38,41,50,51], visual (3/10, 30%) [35,38,51], and haptic (1/10, 10%) [38] cues. A real-time adaptive strategy was used in most of the studies (9/10, 90%) [35-38,40-42,50,51], while 20% (2/10) of the studies used a deferred adaptive strategy [41,43]. Finally, regarding the adaptive engine used, either a non-ML–based method was implemented (8/10, 80%) [35,36,38,40-43,50,51], or an ML-based method was applied (3/10, 30%) [37,51]. The number of studies counted does not equal the total number of studies because some of the studies (2/10, 20%) used different adaptive strategies for level switching and feedback, while others (8/10, 80%) used only one of them.

The studies adapted their system through an explicit behavioral signal, such as task performance (6/10, 60%) [36,40-42,50,51]; or an implicit biosignal, such as motor movements (3/10, 30%) [37,38,51], eye gaze (2/10, 20%) [35,42], speech (1/10, 10%) [43], and peripheral physiological responses (1/10, 10%) [36].

**Table 1:** Characteristics of the selected studies.

Study, year	Sample size (n); age (years), mean (SD)			Study objective		Training focus		Device used		Adaptive strategy					Signal used
	ASD^a^	TD^b^							Level switching		Feedback	Time	Engine		
Bekele et al [35], 2016	6; 15.77 (1.87)	6; 15.20 (1.68)	Treatment, usability		Social skills		Desktop computer		—^c^		Visual	Real time	Non-ML^d^ based	Eye gaze	
Hocking et al [37], 2022	10; 14.1 (2.6)	—; —	Treatment, usability		Motor skills		HMD^e^		Progression		—	Real time	ML based	Motor movements	
Jyoti and Lahiri [50], 2020	20; 8.7 (2.2)	20; 8.7 (2.3)	Usability		Social skills		Desktop computer		Progression		Audio, verbal	Real time	Non-ML based	Task performance	
Jyoti and Lahiri [51], 2022	20; 8.61 (2.03)	—; —	Usability		Social skills		Tablet computer		Progression		Audio, visual, verbal	Real time	Level switching: non-ML based; feedback: ML based	Task performance, motor movements	
Kuriakose and Lahiri [36], 2016	9; 14.10 (2.6)	—; —	Usability		Social skills		Desktop computer		Regression		Verbal	Real time	Non-ML based	Task performance, peripheral physiological responses	
Lahiri et al [42], 2013	8; 16.00 (2.08)	—; —	Usability		Social skills		Desktop computer		Regression		Verbal	Real time	Non-ML based	Task performance, eye gaze	
Moon and Ke [43], 2023	4; 12.25 (0.50)	—; —	Treatment		Social skills		Desktop computer		—		Verbal	Deferred time	ML based	Speech patterns	
Pradeep Raj and Lahiri [40], 2016	2; 16.25 (3.40)	2; 11.9 (2.7)	Usability		Social skills		Desktop computer		Regression		Verbal	Real time	Non-ML based	Task performance	
Vallefuoco et al [41], 2022	10; 11.9 (2.7)	—; —	Treatment, usability		Executive functions		Desktop computer		Progression		Audio, verbal	Level switching: deferred time; feedback: real time	Non-ML based	Task performance	
Zhao et al [38], 2018	6; 9.66 (1.36)	6; 10.73 (1.91)	Usability		Motor skills		Desktop computer		—		Audio, visual; verbal; haptic	Real time	Non-ML based	Motor movements	

^a^ASD: autism spectrum disorder.

^b^TD: typical development.

^c^Not applicable.

^d^ML: machine learning.

^e^HMD: head-mounted display.

## Discussion

### Main Findings

The main findings indicate a trend of balanced use among adaptive strategies such as level switching (with slight preferences toward progression techniques) and feedback (with a preference for verbal mode). Furthermore, a widespread use of non-ML techniques was found in engines that used explicit and implicit signals, with a slight tendency toward the latter. Finally, the results showed significant advantages for real-time adaptations.

Overall, the mean age range of the participants in the selected studies (8-16 y) suggests a tendency to prefer a sample composed of adolescents rather than young children. Extended studies [44,52] highlight the benefits of early intervention (particularly for children younger than adolescents) in achieving significant improvements by targeting the fundamental behavioral and skill deficits associated with ASD. Indeed, younger children tend to make better progress in their treatment, even with lower-intensity programs, than older children [44]. Considering the theoretical foundations and the benefits that early intervention brings to children with ASD, future studies involving adaptive VR systems should consider a younger age sample, as reported in other research fields on ASD treatment, such as interventions using robots [53-55]. In this context, when designing an early intervention, it is crucial to consider pediatric recommendations advocating for a maximum screen time of 1 hour per day for children aged 2 to 5 years because this contributes to establishing a high-quality program [56]. Consequently, the implementation of any technological tools must be carefully crafted and controlled.

Furthermore, concerning study objectives, the findings indicated that most of the studies (9/10, 90%) primarily focused on usability [35-38,40-42,50,51]. This confirms the relatively recent emergence of research in applying adaptive VR systems in ASD interventions [20,23-25]. Indeed, an emerging field must first validate new methods and technologies through usability studies. Moreover, a few of the studies (3/10, 30%) had another clinical goal of validating the designed treatment, which is valuable considering that these are innovative and previously untested adaptive systems [35,37,41]. In addition, the prevalence of training targeting social skills was observed in a majority of the studies (7/10, 70%) [35,36,40,42,43,50,51], aligning with a well-established focus in ASD intervention literature that emphasizes the importance of addressing the social challenges faced by individuals with ASD [57,58].

Another prominent trend observed relates to the device deployed. Notably, among the selected studies, there was a consistent preference for desktop computers and similar devices, such as tablet computers [35,36,38,40-43,50,51], which is consistent with the broader literature on adaptive VR training [5]. The prominent decision to opt for nonimmersive VR devices showcase a practical choice grounded in considerations of accessibility, affordability, and cost-effectiveness, as well as the avoidance of potential discomfort [36,42,50,51,59]. Nevertheless, it is likely that nonimmersive VR systems were chosen because evidence suggests that they could also support individuals with ASD, particularly in addressing comorbidities such as attention-deficit/hyperactivity disorder and anxiety disorders [60,61]. Further research targeting the impact of nonimmersive VR systems on comorbid conditions with ASD would be valuable for elucidating their full potential and applications.

The following subsections are organized as follows: the Adaptive Strategy: Regression, Multimodal, and Real-Time Adaptation subsection delves into findings regarding the adaptive strategy in terms of mode and time; the next subsection, The Adaptive Engine: Non-ML and ML Techniques, delineates the techniques chosen to adapt the engine used; and, finally, the Signals: Embodied Adaptation subsection presents a detailed discussion about the signals involved. Each subsection includes a segment aimed to highlight methodological considerations and guide future research development.

### Adaptive Strategy: Regression, Multimodal, and Real-Time Adaptation

The examination of the adaptive systems in the selected studies reveals a critical interplay between level switching and feedback strategies, both essential components for addressing the challenges associated with individualized training. Nevertheless, not all studies implemented both adaptive strategies in their intervention. Considering the level-switching strategy, in studies using progression-based adaptations [37,41,50,51], the difficulty level was automatically increased after sufficient performance, while the same level was repeated after insufficient performance; for example, Jyoti and Lahiri [50] used a performance threshold score of ≥70% as the criterion for increasing the difficulty level. In this application, the performance score was calculated based on the accuracy in recognizing regions of the virtual character’s face within the maximum response time allowed [50]. In the study by Hocking et al [37], the performance threshold score was raised to ≥75% to continuously update the challenge. However, performance was quantified by calculating the efficiency, synchrony, and symmetry of the movement performed [37]. Contrastingly, studies that adopted the regression technique [36,40,42] increased the level of difficulty after sufficient performance, while the level remained the same or decreased in the case of semi-insufficient or insufficient performance, respectively. Specifically, Kuriakose and Lahiri [36] designed rules to determine whether the difficulty level should increase (performance score ≥70% and anxiety level <6 units or performance score <70% and anxiety level <6 units), remain the same (performance score ≥70% and anxiety level ≥6 units or performance score <70% and anxiety level <6 units), or decrease (performance score <70% and anxiety level ≥6 units or performance score ≥70% and anxiety level ≥6 units) based on the performance score along with the predicted anxiety level. In the study conducted by Pradeep Raj and Lahiri [40], performance was assessed on cognitive and emotional tasks, with adjustments made solely based on the possibility of increasing the difficulty level when performance was sufficient (≥70%) and decreasing it when performance was not sufficient.

In the training context, feedback plays a pivotal role in guiding and enlightening the system’s user regarding their performance, providing responses to both explicit and implicit behaviors. Of the 10 selected studies, 9 (90%) implemented at least 1 feedback mode in their system; the sole exception was the study by Hocking et al [37]. The decision to use one mode over the other seems contingent upon the specific training goals and the nature of the skills being developed. The findings reveal that a majority of the selected studies (8/10, 80%) adopted the verbal feedback mode [36,38,40-43,50,51]. This preference can be attributed to the fact that listening to audio messages or reading written communication allows complex information to be transmitted. Moreover, one-fourth of the studies (4/10, 40%) opted for a combination of feedback modes [38,41,50,51]. In fact, it is well known that multimodal communication is more likely to be received effectively and has a greater informational effect [62]; for example, in the study by Jyoti and Lahiri [51], feedback was provided at the end of each task based on criteria governed by performance and face orientation. The feedback included verbal, visual (ie, the number of stars), and auditory (ie, clapping hands) components, delivered with positive or negative connotations to reinforce behavior. The exploration of multimodal feedback, as demonstrated by Zhao et al [38], introduces a compelling dimension to the discussion by successfully implementing complex multimodal feedback. The incorporation of 4 different feedback modes—verbal (ie, written message), auditory (ie, crash audio and achievement jingle), visual (ie, reward or warning images), and haptic (ie, friction and spring force)—represents a sophisticated approach to enhancing user engagement [38]. The synergistic effect of multiple sensory modalities, as supported by previous literature [63], suggests that integrating diverse feedback elements can have a cumulative positive impact on the user’s learning experience.

This review also delves into the temporal considerations of implementing adaptive strategies. These strategies can be transmitted from the engine to produce an ad hoc adaptive decision either during the session (real time) or between sessions (deferred time). The review’s results indicated a preference for adapting the level or the feedback in real time rather than deferred time [35-38,40-42,50,51]. Real-time adaptation seems promising because it aligns with the potential to enhance the training experience by tailoring content in response to the user’s performance [5]; for instance, Bekele et al [35] designed a stimuli occlusion paradigm in which the stimuli were revealed gradually based on the eye gaze performance of the participants: the more attentively participants turned their gaze toward the stimulus, the clearer and sharper it became—providing visual feedback. A specific case in the study by Vallefuoco et al [41] involved feedback provided in real time based on the accuracy of participants’ actions; here, the difficulty level of the first session was chosen based on the *Diagnostic and Statistical Manual of Mental Disorders, Fifth Edition*, severity levels of ASD symptoms [27], and the difficulty level seemed to progressively increase in line with the game levels and symptom severity. In addition to the duality of real-time (feedback) and deferred (level switching) adaptivity of the 2 strategies, there was further duality inherent in level switching: the initial level was set by the therapist and then automated by the system. Indeed, this study was included in this review because, at first, the system was person automatized and then became system automatized (refer to the next subsection, The Adaptive Engine: Non-ML and ML Techniques), highlighting the advantages of cost-effectiveness and streamlined implementation.

Given these premises, it seems advantageous to introduce at least 1 strategy in real time when designing an adaptive automatized system. Training that uses a scaffolding strategy, which recognizes the need to adjust the level switching up or down as needed (regression technique), has consistently demonstrated better learning outcomes [8]. Similarly, feedback is considered a scaffolding strategy useful for adapting intent and capabilities during the training by promoting critical thinking [64]. Considering the findings, the power of verbal and multimodal feedback [62], and the benefits of in-session administration of the 2 adaptive strategies [5,64], future studies should include progression level switching and real-time verbal feedback in their adaptive interventions. Future research should also carefully consider the interplay between these adaptive strategies and their potential synergistic effects on training efficacy. Practitioners and policy makers involved in designing training interventions should recognize the complementary nature of these strategies and strive to incorporate them synergistically. In conclusion, the comprehensive analysis of adaptive strategies sheds light on their intricate dynamics within individualized training environments. By incorporating these insights into practice frameworks, future researchers can work toward enhancing the effectiveness, accessibility, and scalability of adaptive training interventions across various domains.

### The Adaptive Engine: Non-ML and ML Techniques

A system-automatized adaptive engine can independently analyze signals and decide when to apply level switching or feedback to customize the intervention for each individual. In this context, the deployment of an adaptive engine in the reviewed studies confirms the dichotomy in the techniques used for decision-making: non-ML and ML. A significant majority of the reviewed studies (8/10, 80%) lean toward non-ML techniques for adaptive engine design [35,36,38,40-42,50,51]. This approach involves establishing predefined rules, often binary in nature, to determine the appropriateness of the participant’s performance. Decisions are made through established cutoffs to determine whether the performance is sufficient in terms of duration, successes, gazed area, accurate movements, and physiological activity levels; for instance, Kuriakose and Lahiri [36] designed an adaptive engine based on the composite effect of physiological index levels (high or low) through the fuzzy logic classification method and the quality of task performance (adequate or inadequate). Similarly, Lahiri et al [42] adapted their VR system through complex fixed rules that blended information from viewing patterns (fixation duration), eye physiology (pupil diameter and blink rate), and task performance. The inflexibility of non-ML techniques is evident in their reliance on a priori knowledge and fixed rules. While effective for binary decisions, the limitation lies in their inability to adapt to more intricate data nuances not easily discernible by human observation. In contrast to non-ML techniques, the primary goal of ML techniques is to predict future observations as accurately as possible and increase efficiency and reproducibility [65,66]. Despite the advantages of adopting ML techniques, a limited number of the selected studies (3/10, 30%) opted for ML techniques to drive adaptive engine decisions [37,43,51]. The use of artificial neural networks by Hocking et al [37] exemplifies the capacity of ML techniques to objectively quantify kinematic features by tracking the biomechanical changes and adapting the challenge level in real time. Specifically, the authors applied a normalized exponential transformation translated into a discrete probability assignment over the potential labels and trained this model until no further enhancements were observed [37]. Similarly, Moon and Ke [43] analyzed participants’ speech and used a supervised ML method to categorize the probability values into threshold conditions (from high to low emotional state). Feedback was then tailored based on the individual’s threshold condition. The novel integration of ML and non-ML approaches in a single study, as seen in that by Jyoti and Lahiri [51], showcases the potential for a hybrid model. While the task performance level switching remained non-ML, the use of ML-based feedback signaled a nuanced approach to harnessing the strengths of both techniques [51]. The ML-based feedback relied on image processing using a Haar feature–based cascade frontal face classifier [67] to calculate the percentage of the user’s face (gross motor movement) oriented toward the area of interest. However, the method proposed by Viola and Jones [67] is now considered obsolete because it has been replaced with deep learning methods, such as convolutional neural networks [68,69].

Considering the factors discussed, the distinct characteristics of non-ML and ML techniques raise critical considerations for their application in adaptive systems. Non-ML techniques, which are reliant on predetermined rules, demonstrate efficacy in scenarios where binary decision-making aligns with straightforward performance metrics. However, their limited ability to discern subtle patterns in data necessitates caution when confronted with complex and dynamic user interactions that are usually not detectable by humans. Conversely, ML techniques, driven by continuous objective analysis of data sets and pattern recognition, hold promise in offering adaptive systems that improve automatically over time. Considering a growing emphasis on adaptive systems based on ML [5], the ability to replicate outcomes and accurately predict future observations, as highlighted by Orrù et al [65], positions ML as a potent tool to provide more refined and personalized interventions. Future research should carefully weigh the trade-offs between non-ML and ML approaches. This review advocates for increased adoption of ML techniques in future research aiming to enhance ASD interventions.

However, it is notable that among the selected studies that used ML techniques, the samples ranged from 4 to 20 participants [37,43,51]. In ML models, predictions are made by using versatile learning algorithms to find patterns in typically vast data sets; this is why extensive data sets are necessary to yield more accurate algorithm outcomes [65,66]. Acknowledging that larger data sets enhance the generalization performance of ML models [66], the limited sample sizes in these studies raise concerns about the robustness of the ML-based adaptive engines. Future research should prioritize expanding sample sizes to bolster the reliability and effectiveness of ML-driven interventions.

Among other advantages, the adaptive engine, functioning as the autonomous core of the system, exhibits the capability to make real-time decisions. Regardless of the chosen technique (non-ML or ML), an overarching theme in the reviewed studies is the pervasive use of real-time adaptive systems. This aligns with the growing interest in adaptive systems that use ML techniques to recognize patterns in data, enabling real-time responses and an enhanced user experience [34,39]. This is frequently accomplished using sensors and algorithms for effective data interpretation and analysis, harnessing the input signals to optimize the system’s performance. It is suggested to implement this approach in future studies to enhance the system’s ability to provide meaningful responses based on the gathered dynamic user inputs. Policy makers could support initiatives aimed at advancing sensor technology and algorithm development to facilitate real-time data interpretation and analysis in adaptive systems, which could enhance their effectiveness in clinical settings.

In conclusion, the architectural choices for adaptive engines play a pivotal role in shaping intervention efficacy. The synthesis of real-time adaptability and a preference toward adopting non-ML techniques as well as a consideration of the nuances of sample size in ML-based approaches should guide future endeavors in developing adaptive systems that seamlessly integrate cutting-edge technologies to enhance user experiences in ASD interventions.

### Signals: Embodied Adaptation

Given what has been discussed with respect to the adaptive strategies governed by the engine and the techniques used by the engine, it is pertinent to address what drives and prompts these adaptation decisions: signals.

The results of this review showed a similar reliance on explicit and implicit signals, with a slight preference for the latter, in shaping ASD training adaptations. In particular, a third of the studies (3/10, 30%) [40,41,50] exclusively used explicit signals, focusing on behavioral data such as task performance metrics (ie, reaction time and success rates). Another set of studies (3/10, 30%) [36,42,51] adopted a more integrated approach, combining explicit signals (task performance) with implicit biosignals such as gross motor movements, peripheral physiological responses, and eye gaze. Jyoti and Lahiri [50,51] highlighted the importance of adapting training through an implicit measure complementary to performance data. Indeed, in their latest study [51], the authors introduced informative data on the duration of face orientation toward the stimulus presented to infer whether the participant was attending to the task stimulus. Adding implicit biosignals might augment the potential of explicit signals, leading to more valid outcomes. Similarly, Kuriakose and Lahiri [36] adapted their VR system to users’ affective states, such as anxiety, through peripheral physiological signals (including galvanic skin response and pulse plethysmogram). Adaptive training based on implicit biosignals has been demonstrated to hold the potential to optimize physiological arousal to maximize training outcomes [5]. Conversely, training programs that fail to adjust to users’ affective states may negatively impact performance, leading to either low arousal levels (as in boredom) or high arousal levels (as in anxiety) [29]. To prevent this, it is essential to consider both implicit and explicit biosignals. A combined analysis of users’ implicit and explicit biosignals enables refined adaptation decisions based on their needs. During treatment, implicit biosignals can provide valuable insights into the user’s inner state, which is often imperceptible to human observation but easily deciphered by computers [36]. Indeed, compared with explicit signals, implicit biosignals emerge as pivotal in providing optimal challenges to users, increasing their engagement and improving their performance [29,30]. The study by Lahiri et al [42] stands out as a pioneering work, in comparison with the other included studies, in anticipating the benefits of adapting training or interventions through implicit biosignals. Indeed, it proposed measuring the engagement level through real-time viewing patterns, subtle eye physiological changes, and performance metrics to adaptively enhance responses for improved social communication skills. This underscores the clear need to incorporate an implicit biosignal when training or investigating a complex construct.

Recent studies have suggested the integration of advanced technologies (eg, eye gaze monitoring, peripheral response monitoring, motion sensing, and speech input devices) to capture implicit biosignals, thereby steering ASD interventions toward a more intuitive and embodied approach [29,35,40]. As discussed, implicit biosignals unveil hidden meanings and provide insights that extend beyond observable user behaviors. This aligns with the concept of embodied cognition [70], which posits that body and mind are not separate entities but rather maintain a continuous, tight, and ongoing relationship. Embodied cognition theories are based on the concept that every action or reaction is influenced by one’s perception of stimuli, typically during goal achievement [70,71]. VR interventions, as embodiments of learning paradigms, represent an illustration of how technology links the mind, body, and environment together and where individuals’ perceptions play a crucial role in shaping their bodily experience of the world [72]. The selected studies in this review reflect a growing body of evidence supporting the adaptation of interventions to the unique needs of children with ASD, incorporating devices capable of collecting implicit biosignals of fine [38] or gross [37,51] motor movements, eye gaze [35,42], peripheral physiological responses [36], and speech [43]; for example, Moon and Ke [43] used speech data mining to drive adaptive feedback to identify the emotional states of children.

On the basis of the assumption that embodied cognition theories indicate that cognition involves a continuous interplay of various modules encompassing simulation, environmental, situated action, and bodily state factors [73], implicit biosignals are at the core of the process of cognition because they reflect and explain behaviors that are not directly observable. Thus, future adaptive interventions for children with ASD should go beyond monitoring user performance as a stand-alone datum and adopt an embodied perspective by including implicit aspects, assuming that the arousal component influences the learning experience in training. Cognition rarely proceeds independently of the body; however, it is recognized that many researchers address other forms of cognition assumption [70]. Therefore, the suggestion is to implement the embodied adaptation framework but acknowledge that other adaptations may also be valuable. Practitioners could support initiatives aimed at promoting embodied perspectives in ASD interventions, recognizing their potential to enhance learning experiences and outcomes for individuals with ASD.

Finally, attention should be focused on those included studies (6/10, 60%) that processed implicit biosignals in real time to enhance ASD adaptive interventions [35-38,42,51]. Evidence suggests that VR-automatized systems, which adapt dynamically based on implicit biosignals, are able to foster ongoing communication with users by identifying their present state and aligning activities with specific behavioral objectives [37,38]; for example, sophisticated algorithms have made it possible to quantify and qualify dynamic whole-body motor movement in real time, allowing for the adaptation of motor skills interventions [37]. Even finer motor movements, such as force or precision and control, can be adapted in real time to improve the performance of a kinetic task [38]. In conclusion, the spotlight on studies processing explicit and implicit biosignals in real time underscores their potential to enhance the effectiveness of adaptive interventions for young individuals with ASD. Moreover, as technology continues to advance, the integration of unobtrusive wearable devices that capture implicit biosignals, either independently or in combination with explicit signals, emerges as a promising approach [5]. On the basis of the literature and the reasons discussed, it is recommended to embrace the embodied cognition perspective to comprehensively understand and effectively address the unique needs of children with ASD.

### Overall Findings

The literature search showed that there has been a growing emphasis on developing objective adaptive technologies to target the needs of individuals with ASD. This review primarily aimed to shed light on the methodologies of the included studies used for the adaptive engine and the types of signals used. In addition, the review identified methodological insights and offered guidance for improving adaptive VR interventions for individuals on the autism spectrum.

The findings suggested a tendency to focus on adolescent samples, underscoring the need to explore younger age groups in the context of adaptive VR systems. A predominant focus on usability, social skills training, and the use of desktop computers was discussed.

The main findings showed that the use of adaptive strategies varied among studies, with a noteworthy trend toward real-time adaptations. The nuanced application of progression techniques, coupled with the exploration of verbal feedback and real-time adaptations, provides a foundation for future research aimed at optimizing adaptive training systems for therapeutic contexts.

The review highlighted considerations associated with non-ML and ML techniques critical in the engine adaptation. An overarching theme in the reviewed studies is the widespread use of real-time adaptive systems, mostly non-ML, although there has been a growing emphasis over the past 2 years on using ML techniques for refined and enhanced training. Moreover, when choosing an ML engine, a wider sample size should guide the development of future adaptive systems for personalized ASD interventions.

Finally, both explicit and implicit signals were identified as the driving force behind adaptive decisions, with increased attention toward using implicit biosignals for a more comprehensive understanding of user needs. Embracing an embodied adaptation framework is suggested for future ASD interventions, emphasizing real-time processing to optimize adaptation and contribute to improved performance.

By synthesizing and evaluating the existing evidence, this study aims to contribute valuable insights that can inform the design and implementation of more targeted and effective interventions, ultimately enhancing the quality of life for individuals with ASD and their families [74]. In fact, an adaptive system holds the promise of improving outcomes for greater numbers of children with ASD by guiding clinicians on how to leverage the diversity in treatment responses [6].

### Limitations

This review is not without limitations. No technological or automated tools were used to handle the collected data, which could have introduced bias due to human error. However, because the literature search identified only a small number of articles, manual analysis was feasible, and it did not become necessary to use specific automated techniques. The limitations of this review primarily stem from the heterogeneity in the study designs of the selected studies. Future reviews should focus on different inclusion and exclusion criteria with more consistent designs to reduce variation and improve comparability. In addition, the availability of data in the selected studies posed another constraint. Some of the studies (3/10, 30%) lacked sufficient information regarding the adaptation of their systems, making it challenging to conduct specific analyses or fully assess the study’s contribution.

### Conclusions

In recent years, the field of ASD interventions has witnessed a significant shift toward the development of objective adaptive technologies. This evolving paradigm aims to not only enhance the overall effectiveness of interventions but also customize them to better suit the unique needs of individuals with ASD. A systematic review was conducted to assess the current body of literature concerning the application of adaptive VR systems in ASD interventions.

The findings showed trends that align with theories and similar studies focused on broader themes, such as the significant interplay between level-switching strategies and feedback and a preference for adaptive engines operating in real time [5,64]. However, throughout the paper, we also offered several reflections on how the methodological focus seems to be shifting. Specifically, we observed how implementing multimodal feedback could enhance adaptive interventions in terms of both qualities and opportunities [38,62,63]. We have provided critical insight concerning the growth of adaptation studies implementing ML techniques. Indeed, among the included records, those from the past 2 years have primarily focused on this technique (3/10, 30%) [37,43,51], likely due to its versatile and accurate nature [65,66]. Similarly, the importance of implicit biosignals was rationalized within the theoretical framework of embodied cognition [72,73] for a deeper understanding of unobservable behaviors.

This work can support researchers in designing and testing adaptive VR systems for ASD interventions and help software designers develop more engaging and targeted VR applications, thus improving the effectiveness of digital therapies. In addition, it can contribute to the research of innovative methodologies for assessing and monitoring progress in ASD treatments using advanced VR tools. It can also provide valuable support to clinical professionals in better adapting interventions to patients’ states and specific needs. Finally, it can guide practitioners toward a deeper understanding of the potential of VR in ASD treatments, positively influencing decision-making in the formulation of health care and educational policies.

## Appendix

Multimedia Appendix 1 PRISMA (Preferred Reporting Items for Systematic Reviews and Meta-Analyses) checklist.

## Abbreviations

ASD: autism spectrum disorder
ML: machine learning
PRISMA: Preferred Reporting Items for Systematic Reviews and Meta-Analyses
VR: virtual reality
